# Low expression of RecQ-like helicase 5 is associated with poor prognosis in patients with gastric cancer

**DOI:** 10.3892/ol.2019.11137

**Published:** 2019-11-21

**Authors:** Yijia Lin, Huashe Wang, Xinyou Wang, Miao Li, Honglei Chen, Junsheng Peng

**Affiliations:** 1 Department of Gastrointestinal Surgery, Guangdong Provincial Key Laboratory of Colorectal and Pelvic Floor Diseases, The Sixth Affiliated Hospital, Sun Yat-sen University, Guangzhou, Guangdong 510655, P.R. China; 2 Department of Digestion, Guangdong Provincial Key Laboratory of Colorectal and Pelvic Floor Diseases, The Sixth Affiliated Hospital, Sun Yat-sen University, Guangzhou, Guangdong 510655, P.R. China; 3 Gastrointestinal Endoscopy Center, The Eighth Affiliated Hospital, Sun Yat-sen University, Shenzhen, Guangdong 518033, P.R. China

**Keywords:** RecQ-like helicase 5, gastric cancer, immunohistochemistry, prognosis

## Abstract

The role of RecQ-like helicase 5 (RECQL5) in gastric cancer (GC) is unclear. This study investigated the expression, clinicopathological association and prognosis of RECQL5 protein in human GC. Firstly, the Oncomine database was used to determine the mRNA expression levels of RECQL5 in GC samples. GC samples and adjacent normal gastric tissue samples were subsequently assessed to determine RECQL5 protein expression levels using immunohistochemistry. The clinicopathological association with RECQL5 expression was analyzed. Multivariate Cox analysis was performed to determine the relationship between RECQL5 expression and survival outcomes. Data from the Oncomine database revealed that RECQL5 mRNA was significantly downregulated in GC tissues compared with that in normal gastric tissues (P<0.05). These results were then validated at the protein level as RECQL5 protein expression was found to be significantly downregulated in GC samples compared with that in normal gastric tissues (P<0.05). Low expression of RECQL5 was significantly associated with depth of tumor invasion, histological differentiation and TNM stage (all P<0.05) and indicated poor prognosis in patients with GC. Multivariate analysis revealed that low RECQL5 expression and depth of invasion were independent prognostic factors for GC (P<0.05). These results suggest that low expression of RECQL5 is associated with carcinogenesis and invasion in GC and with poor overall survival in patients with GC. RECQL5 may be a novel prognostic marker for patients with GC.

## Introduction

Gastric cancer (GC) has been considered as a common primary malignant tumor with the fourth-highest cancer-related mortality rate worldwide in the last decade (1). In China in 2015, ≥60% of patients with GC had advanced disease (2). In the last decade, with the development of GC therapy, many cancer markers such as programmed death-ligand 1 and human epidermal growth factor receptor 2 have been assessed as candidate prognostic factors and therapeutic targets for GC (3,4). The 5-year survival rate and quality of life of patients with GC has improved markedly (5–7). However, patients in the advanced stage still have a poor prognosis, and advanced GC poses a higher burden for patients and society (8). Thus, markers that accurately predict the prognosis of patients with GC are needed.

RecQ helicases play a critical role in maintaining genome stability, as well as DNA recombination, replication and transcription (9–11). There are five RecQ helicases in human cells: RecQ-like helicase 1 (RECQL1), Werner syndrome RecQ-like helicase (WRN), Bloom syndrome RecQ-like helicase (BLM), RecQ-like helicase 4 (RECQL4) and RecQ-like helicase 5 (RECQL5) (9,10,12) Mutations in WRN, BLM and RECQL4 proteins can lead to genomic instability and predisposition to cancers, including colorectal, prostate and breast cancers (13–16). Important roles of RECQL5 have been identified in DNA replication and transcription, base excision repair and homologous recombination (17,18). Lao *et al* (19) reported the abrogation of RECQL5 expression in colorectal cancer. Another study demonstrated that RECQL5 acts as a tumor suppressor in osteosarcoma, and increased expression of RECQL5 can inhibit the progression of osteosarcoma (20). Conversely, other studies showed that RECQL5 is overexpressed in breast cancer and bladder carcinoma, and that depletion of RECQL5 can significantly reduce the progression of cancer (21,22). However, the roles of RECQL5 in GC remains unclear.

In the present study, expression of RECQL5 was investigated by mining the publicly available Oncomine database, combined with validation in samples from patients with GC and normal adjacent tissues using immunohistochemistry. The clinicopathological and prognostic significance of RECQL5 in patients with GC was also evaluated.

## Materials and methods

### 

#### Bioinformatics prediction

The RECQL5 mRNA data from GC and normal gastric tissues were extracted from the Oncomine online database (https://www.oncomine.org). The filtered datasets were analyzed separately. RECQL5 expression values between normal gastric tissues and GC tissues were extracted and compared from the Chen Gastric, DErrico Gastric and Cho Gastric datasets (23–25). The Kaplan-Meier plotter online (http://kmplot.com/analysis/) was used to predict the overall survival (OS) outcomes of patients with GC (26). This software contains a public database of Affymetrix microarray data from 1,065 patients with GC (ID, 211468_s_at). To analyze the prognostic value of RECQL5 in GC, the samples in the database were divided into 2 groups: High and low expression of RECQL5. The relationship between RECQL5 expression and survival data was analyzed using Kaplan-Meier survival curves. The log rank P-value and hazard ratio (HR) with 95% confidence intervals (CIs) were calculated.

#### GC tissue specimens and clinicopathological data

Informed consent was obtained from all individual participants included in the study, and the specimens were collected after approval from the Institute Research Medical Ethics Committee of The Sixth Affiliated Hospital, Sun Yat-sen University (Guangzhou, China). A total of 78 cancer specimens (age range, 38–76 years) were collected from patients with GC and matched with adjacent normal gastric tissues. The distance between tumor and normal tissues was >1 cm. The patients with GC underwent radical surgery between January 2009 and August 2011 at the Sixth Affiliated Hospital, Sun Yat-sen University (Guangzhou, China).

#### Immunohistochemistry

Paraffin embedded sections were used for immunohistochemistry. The thickness of the slides was 4 µm. Biotin-Streptavidin HRP Detection Systems (cat. no. SP-9001; Beijing Zhongshan Golden Bridge Biotechnology Co., Ltd.) was used to detect RECQL5 expression in GC samples. Staining was performed according to an established protocol using a rabbit polyclonal antibody against human RECQL5 (Sigma-Aldrich; Merck KGaA; cat no. HPA029971) diluted in PBS (1:150). Slides were incubated at 4°C in a moist chamber overnight with the primary antibody. Slides stained with PBS instead of primary antibody were used as negative controls. The visual immunoreactivity score (IRS) was calculated by using the following formula: Staining intensity (SI) × percentage of stained cells with that intensity. IRS values were used to determine the expression level of RECQL5. The SI scores were as follows: 0, negative; 1, weak; 2, moderate; and 3, strong. The percentage of stained cells was calculated as the percentage of positively-stained tumor cells in the field, and was expressed as follows: 0, negative; 1, 0–25%; 2, 26–50; 3, 51–75%; and 4, >76%. Based on the SI scores, the RECQL5 expression level was classified as high (grades 4–12) or low (grades 0–3). Patients were classified into 2 groups, RECQL5 high and low. The tissues were independently scored by 2 pathologists who were blinded to the origin of each tissue. For any discrepancy, the 2 pathologists reassessed the slides together to reach an agreement.

#### Statistical analysis

SPSS version 22.0 (IBM Corp.) was used for statistical analyses. Ordinary one-way ANOVA was used to analyze the expression difference of RECQL5 from the Oncomine database. The association between clinicopathological features and RECQL5 protein expression was assessed using a χ^2^ test. The survival rate was assessed using Kaplan-Meier curves and the log-rank test. Cox proportional hazards regression model was applied for multivariate analysis to determine independent prognostic factors of GC. P<0.05 was considered to indicate a statistically significant difference.

## Results

### 

#### RECQL5 mRNA and protein expression is low in patients with GC

**Figure 1.**
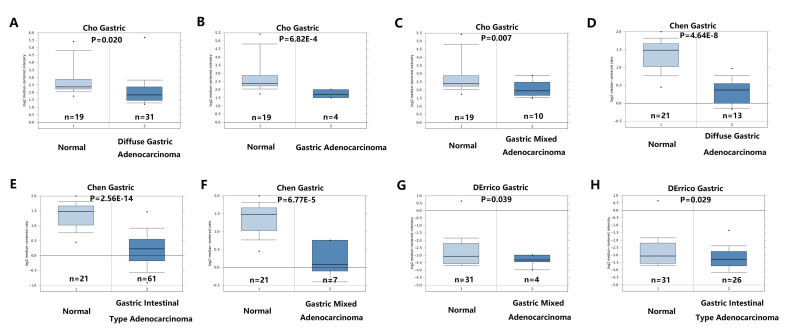
Expression of RECQL5 mRNA in human gastric cancer and normal gastric tissue using the Oncomine database. RECQL5 mRNA expression in (A-C) Cho Gastric dataset; (D-F) Chen Gastric dataset; (G and H) DErrico Gastric dataset. RECQL5, RecQ-like helicase 5.

Oncomine database analysis demonstrated that RECQL5 mRNA was downregulated in GC tissues compared with normal gastric tissues. The Cho Gastric dataset indicated that expression of RECQL5 was downregulated in diffuse gastric adenocarcinoma (n=31; P=0.020), gastric adenocarcinoma (n=4; P=6.82×10^−4^), and gastric mixed adenocarcinoma (n=10; P=0.007) compared with that in normal gastric tissues (n=19; Fig. 1A-C). The Chen Gastric dataset revealed low expression of RECQL5 in diffuse gastric adenocarcinoma (n=12; P=4.64×10^−8^), gastric intestinal type adenocarcinoma (n=63; P=2.56×10^−4^), gastric mixed adenocarcinoma (n=8; P=6.77×10^−5^) and compared with normal gastric tissues (n=26; Fig. 1D-F). The DErrico Gastric dataset revealed that RECQL5 was downregulated in gastric mixed adenocarcinoma (n=4; P=0.039), gastric intestinal type adenocarcinoma (n=26; P=0.029) compared with normal gastric tissues (n=31; Fig. 1G and H).

**Figure 2.**
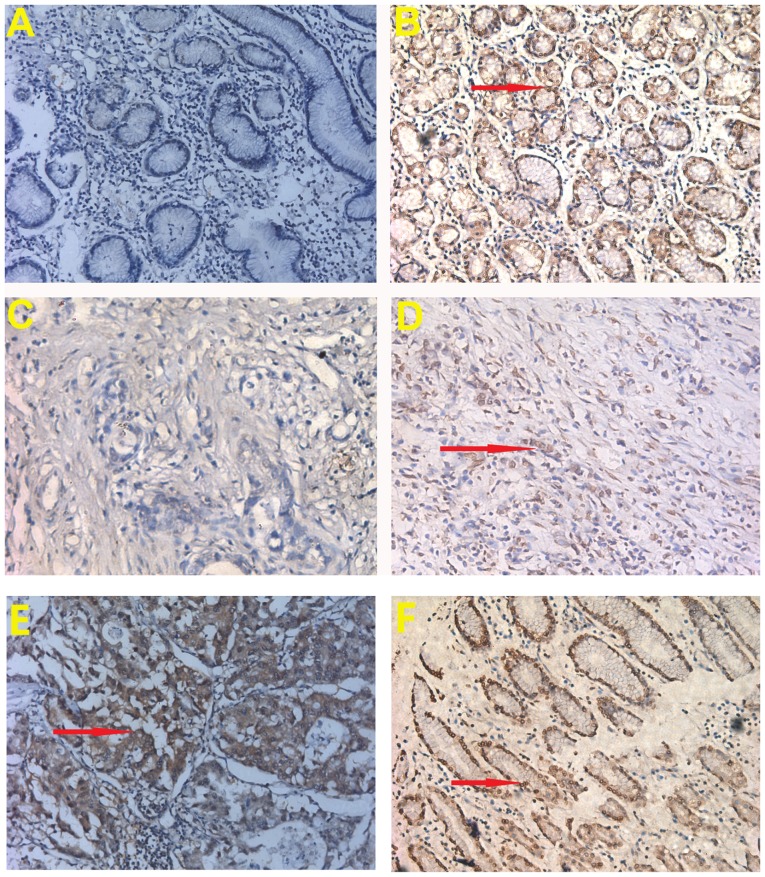
Immunohistochemical staining of RECQL5 in (A) normal gastric mucosal tissues with low expression of RECQL5; (B) normal gastric mucosal tissues with high expression of RECQL5; (C) GC samples with low expression of RECQL5; (D) GC samples with high expression of RECQL5 (poorly differentiated); (E) GC samples with high expression of RECQL5 (moderately differentiated); (F) GC samples with high expression of RECQL5 (well differentiated). The arrows represent RECQL5 protein expression. GC, gastric cancer; RECQL5, RecQ-like helicase 5.

Immunohistochemistry was used to verify RECQL5 protein expression in GC and normal tissues. Expression of RECQL5 was found in GC tissues (Fig. 2D and E). Overall, 71.8% (56/78 samples) of patients with GC displayed low RECQL5 expression in GC samples, while 28.2% (22/78) displayed high RECQL5 expression. In the matched normal gastric tissues, 32.1% (25/78) of the patients displayed low RECQL5 expression and 67.9% (53/78) of the patients had high RECQL5 expression. The results indicated that RECQL5 expression was downregulated in GC tissues compared with normal gastric tissues (P<0.05; Table I), consistent with the results of RECQL5 mRNA expression from the Oncomine database.

#### Association of RECQL5 differential expression and clinicopathological parameters of patients with GC

Low expression of RECQL5 was associated with depth of tumor invasion, histological differentiation and TNM stage (P<0.05), but not with patient age or sex, tumor size, lymph node metastasis, venous or lymphatic invasion or distant metastasis (P>0.05; Table II).

#### Low expression of RECQL5 predicts poor prognosis in patients with GC

**Figure 3.**
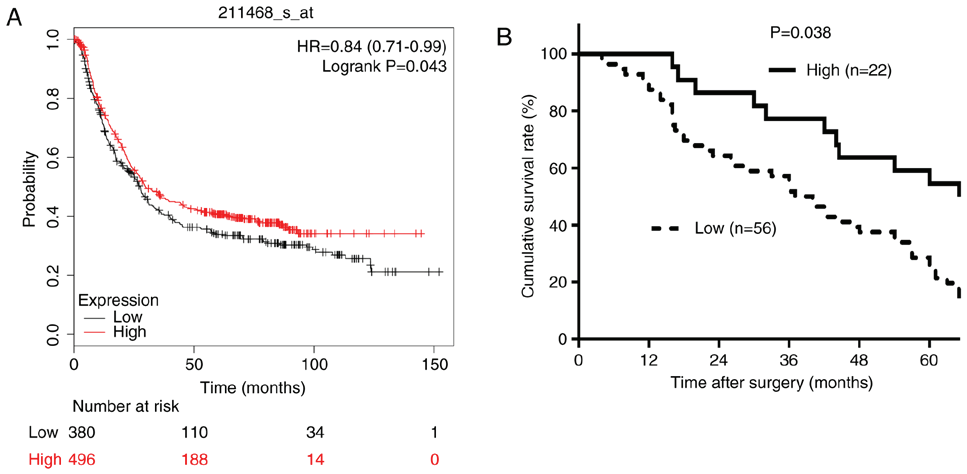
Association between RECQL5 expression levels and OS of patients with GC. (A) OS of patients with GC using the Kaplan-Meier plotter; (B) OS of patients with GC based on immunohistochemical staining. OS, overall survival; GC, gastric cancer; RECQL5, RecQ-like helicase 5.

The association between RECQL5 mRNA expression levels and OS time in patients with GC was investigated using the Kaplan-Meier plotter software. Patients with a low expression of RECQL5 had a shorter OS time (HR, 0.84; 95% CI,0.71–0.99; P=0.043; Fig. 3A). The prognostic value of RECQL5 expression in GC was confirmed using the prognosis data of patients with GC from the Sixth Affiliated Hospital, Sun Yat-sen University (Guangzhou, China). The follow-up time ranged between 4 months and 9.5 years. The 5-year OS rate was 48.7% (38/78 patients). The 5-year OS rate of the RECQL5-low and RECQL5-high groups was 63.6% (14/22) and 42.9% (24/56), respectively. Patients in the low RECQL5 expression group had a significantly shorter OS time (P=0.038; Fig. 3B). The prognosis data was consistent with results from the Kaplan-Meier analysis. Multivariate analysis indicated that the independent prognostic factors were low expression of RECQL5 and depth of invasion (P<0.05; Table III).

## Discussion

Defects of WRN, BLM and RECQL4 may increase cancer predisposition in humans (10). However, whether RECQL5 is associated with cancer predisposition syndrome is unclear. Previous studies have shown that RECQL5 is an essential factor for maintenance of genomic stability, and that RECQL5 may act as an oncogene in various types of cancer (20–22,27). Hu *et al* (28,29) reported that RECQL5 regulates homologous recombination in mouse embryonic stem cells and downregulates the expression of RECQL5 in mice, which can increase susceptibility to colon carcinoma. Lao *et al* (19) demonstrated that loss of RECQL5 expression contributes to the pathogenesis of colorectal cancer. RECQL5 expression is also downregulated in osteosarcoma, and can inhibit proliferation and promote apoptosis of osteosarcoma cells (20). Conversely, a tumor-promoting function of RECQL5 was reported by several studies. Arora *et al* (21) reported the upregulation of RECQL5 in breast cancer due to gene amplification and described a critical role for RECQL5 in cancer progression and demonstrated that small interfering RNA-mediated knockdown of RECQL5 can significantly inhibit *in vivo* tumorigenicity and *in vitro* clonogenic survival of breast cancer cells (30). Patterson *et al* (22) identified a positive association between upregulated expression of RECQL5 with invasion of human urothelial bladder carcinoma. However, RECQL5 in patients with GC has not been fully investigated in previous studies.

In the present study, RECQL5 expression at the mRNA and proteins levels was significantly lower in GC tissues compared with normal gastric tissues. RECQL5 was expressed in 28.2% of GC samples and 67.9% of matched normal gastric tissues. RECQL5 was localized mainly in the nucleus, similar to other studies (19,20). In addition, the low expression of RECQL5 protein was associated with poor histological differentiation, deep tumor invasion and high tumor stage, indicating a prognostic role for RECQL5 in preventing GC progression. Furthermore, patients with high expression of RECQL5 had a higher 5-year OS rate compared with patients with low expression. Thus, low expression of RECQL5 might be a potential prognostic factor in GC. This was verified by multivariate analysis, which indicated that low expression of RECQL5 is an independent marker of poor prognosis, strengthening the hypothesis that RECQL5 may play an important role in preventing the progression of GC. The collective results of this study indicate that low expression of RECQL5 may be a predictor of poor prognosis in patients with GC.

RECQL5 is essential for maintaining genome stability and reducing cancer risk (28). RECQL5 has a tumor-suppressive role in the mouse gastrointestinal tract (29). The results of the present study indicate that the RECQL5 gene may be a candidate tumor suppressor gene in the stomach, and that high expression of RECQL5 may limit tumor growth. The present study also demonstrated that RECQL5 expression was high in normal gastric tissues, which may indicate that RECQL5 plays a role in maintaining genome stability and reducing cancer risk in the stomach. Moreover, low expression of RECQL5 may be a predictor of poor prognosis in patients with GC, which is consistent with previous reports (29,30).

The present study has several limitations. This is a preliminary small-scale bioinformatics and clinical study. As the patient and normal samples were collected non-sequentially from a single center, a selection bias may exist in the study, which may have influenced the findings. Thus, further large-scale studies are required to validate the findings of the present study. In addition, in this study, only the expression of RECQL5 was investigated, and therefore, detailed studies to understand the molecular mechanisms of RECQL5 in GC are required. In conclusion, downregulation of RECQL5 was observed in GC samples. Low expression of RECQL5 was indicative of a more aggressive disease and might be an independent factor of poor prognosis in patients with GC.

## Figures and Tables

**Table I.** Expression of RECQL5 in normal gastric mucosa and primary gastric cancer tissues.

		Expression of RECQL5		
			
Samples	Patients, n	Low, n (%)	High, n (%)	P-value
Gastric cancer	78	56 (71.8)	22 (28.2)	<0.001
Normal gastric tissue	78	25 (32.1)	53 (67.9)	

RECQL5, RecQ-like helicase 5.

**Table II.** Association between RECQL5 expression and clinicopathological features of patients with gastric carcinoma.

		RECQL5 protein expression		
			
Characteristics	Patients, n	Low, n (%)	High, n (%)	P-value
Sex				0.203
Male	48	32 (66.7)	16 (32.3)	
Female	30	24 (80.0)	6 (20.0)	
Age, years				0.240
≥60	45	30 (66.7)	15 (33.3)	
<60	33	26 (78.8)	7 (21.2)	
Tumor size, cm				0.094
≥5	31	19 (61.3)	12 (38.7)	
<5	47	37 (78.7)	10 (21.3)	
Histological differentiation				0.002
Well, moderate	29	15 (51.7)	14 (48.3)	
Poorly, others	49	41 (83.7)	8 (16.3)	
Depth of tumor invasion				0.019
T1-T2	30	17 (56.7)	13 (43.3)	
T3-T4	48	39 (81.3)	9 (18.8)	
Lymphatic invasion				0.957
Yes	28	20 (60.7)	8 (39.3)	
No	50	36 (78.0)	14 (22.0)	
Vascular invasion				0.881
Yes	30	22 (73.3)	8 (26.7)	
No	48	34 (70.8)	14 (29.2)	
Lymph node metastases				0.060
N0	33	20 (60.6)	13 (39.4)	
N1, N2	45	36 (80.0)	9 (20.0)	
Distant metastasis				0.571
M0	53	37 (69.8)	16 (30.2)	
M1	25	19 (76.0)	6 (24.0)	
TNM stage				0.002
I–II	32	17 (53.1)	15 (46.9)	
III–IV	46	39 (84.8)	7 (15.2)	

RECQL5, RecQ-like helicase 5; TNM, Tumor-Node-Metastasis.

**Table III.** Multivariate Cox regression analysis in patients with gastric cancer.

Parameter	P-value	Hazard ratio	95% CI
RECQL5, low vs. high	0.002	2.922	1.504–5.679
Histological differentiation, well, moderate vs. poorly, others	0.326	1.533	0.653–3.598
Depth of tumor invasion, T1-T2 vs. T3-T4	0.019	0.463	0.243–0.880
TNM stage, I–II vs. III–IV	0.544	0.772	0.336–1.778

CI, confidence interval; RECQL5, RecQ-like helicase 5.

## Data Availability

The datasets used and/or analyzed during the present study are available from the corresponding author on reasonable request.
